# A Measure of Resistance: Detecting Tamiflu Metabolite in Sewage Discharge and River Water

**DOI:** 10.1289/ehp.118-a34a

**Published:** 2010-01

**Authors:** Tanya Tillett

**Affiliations:** **Tanya Tillett**, MA, of Durham, North Carolina, is a staff writer/editor for *EHP*. She has been on the *EHP* staff since 2000 and has represented the journal at national and international conferences

During the flu season each year, combating the different strains of the influenza virus becomes a public health priority, with treatment dependent upon two groups of antiviral drugs: neuraminidase inhibitors and M2 ion channel inhibitors. Oseltamivir phosphate, marketed as Tamiflu, is a popular neuraminidase inhibitor widely used to treat flu symptoms. Oseltamivir carboxylate (OC), Tamiflu’s active metabolite, is known to withstand activated sludge treatment at sewage treatment plants (STPs), but less is known about how much OC may make its way into waterways that receive STP effluent. Now a new study conducted in Kyoto City, Japan, during the 2008–2009 flu season reports some of the first measurements of OP occurrences in STP discharge and in river water **[*EHP*** **118:103–107; Ghosh et al.]**.

According to the World Health Organization, between 250,000 and 500,000 people die each year from influenza, and each year, millions of people take Tamiflu to battle flu symptoms. After the sewage treatment process, the excreted active metabolite remains in STP effluent and travels to waterways where effluent is discharged.

The investigators in the current study collected samples from STP effluent and from river water on three different occasions: at the beginning of the flu season, during the peak period, and 2 weeks after the peak period. Using solid-phase extraction followed by liquid chromatography–tandem mass spectrometry, they measured the highest concentration of OC, 293.3 ng/L, in an STP discharge sample collected during the peak of the flu season. Concentration amounts were higher in effluent from STPs that used traditional activated sludge treatment; in contrast, effluent from plants that used advanced ozonation as tertiary treatment contained significantly lower OC levels (37.9 ng/L). River water samples showed a range of OC levels from 6.6 to 190 ng/L during the peak of the flu season.

Previous research indicates OC concentrations ranging from 80 to 230 ng/L will disable 50% of the influenza virus. This level of exposure is most likely to kill virus particles that are particularly susceptible to OC, while selecting for viruses that are resistant to the drug’s effects. The authors note, “During a common flu season, waterfowl can ingest large quantities of OC with virus. . . . Exposing waterfowl infected with influenza A virus to elevated levels of OC in open waterways could trigger the development of Tamiflu-resistant viral strains.”

The authors suggest ozonation as tertiary treatment that may reduce OC load in STP effluent. They also recommend conducting further investigations to determine the fate of antiviral drugs at every interval of the STP process.

## Figures and Tables

**Figure:**
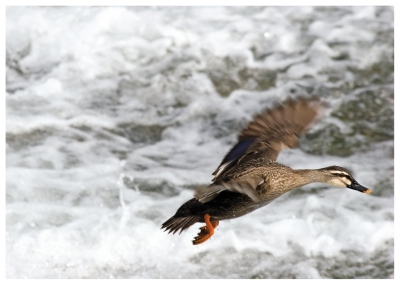
Resistance in the making? Potential influenza A host meets potential source of active Tamiflu metabolite

